# Shape and Temperature Expansion of Free Volume Holes in Some Cured Polybutadiene-Polyisoprene Rubber Blends

**DOI:** 10.3390/ijms22031436

**Published:** 2021-02-01

**Authors:** Giovanni Consolati, Eros Mossini, Dario Nichetti, Fiorenza Quasso, Giuseppe Maria Viola, Erkin Yaynik

**Affiliations:** 1 Department of Aerospace Science and Technology, Politecnico di Milano, Via LaMasa, 34, 20156 Milano, Italy; fiorenza.quasso@polimi.it (F.Q.); giuseppemaria.viola@mail.polimi.it (G.M.V.); erkinyaynik@gmail.com (E.Y.); 2 INFN, Sezione di Milano, Via Celoria, 16, 20133 Milano, Italy; 3 Department of Energy, Politecnico di Milano, Piazza Leonardo da Vinci, 32, 20133 Milano, Italy; eros.mossini@polimi.it; 4 Rheonic Lab, Via Quadelle 2C, 26012 Castelleone, Italy; dario_nichetti@yahoo.it

**Keywords:** dilatometry, elastomers, free volume, positron annihilation lifetime spectroscopy

## Abstract

The free volume fraction of a macromolecular structure can be assessed theoretically by using a suitable model; however, it can also be evaluated from experimental data obtained from dilatometry and positron annihilation lifetime spectra. In this second case, a regular geometry of the sub-nanometric cavities forming the free volume has to be assumed, although in fact they are irregularly shaped. The most popular approach is to guess spherical holes, which implies an isotropic growth of these last with temperature. In this work, we compared the free volume fraction, as obtained from experiments in a set of polybutadiene and polyisoprene cured rubbers and their blends, with the analogous quantity expected by using the lattice-hole model. The results allowed us to obtain insights on the approximate shape of the holes. Indeed, a cylindrical flattened geometry of the cavities produced a better agreement with the theory than the spherical shape. Furthermore, the best fit was obtained for holes that expanded preferentially in the radial direction, with a consequent decrease of the aspect ratio with temperature.

## 1. Introduction

Although not univocally defined [1], the free volume is a useful concept to explain various features of polymers such as mechanical properties [2], diffusivity of permeants [3], or aging [4]. Therefore, the evaluation of the free volume is important not only for basic science, but also to design polymers with tailored properties for specific applications. We can theoretically evaluate a free volume fraction *h* by using the Simha–Somcynsky hole model [5], which has been successfully tested on several amorphous polymers at equilibrium [6]. For this purpose, the equation of state, as derived from the theory, is fitted to dilatometric data versus temperature for the polymer under investigation. Scaled thermodynamic parameters are derived, which allow for the free volume fraction to be found from a mastercurve [7].

From an experimental point of view, we can also determine the free volume as the product between the average volume of a hole and the number density of holes. Since the cavities forming the free volume have typically sub-nanometric sizes, it is not possible to observe them directly and indirect techniques have to be used. Among them, we recall small angle x-ray diffraction [8], the use of photochromic labels [9], photoisomerization [10], and positron annihilation lifetime spectroscopy (PALS) [11]. This last method has gained popularity due to its non-destructive character; furthermore, permanent external probes are not used such as in [10], which may imply some systematic bias in the evaluation of the free volume. PALS uses positronium, Ps, an unstable bound system, which may be formed when a thermalized positron diffusing in the sample under investigation encounters an electron. Ps shares the same size as hydrogen, but it is about two thousand times lighter. Due to the exchange repulsion between the Ps electron and the surrounding ones, the atom is repelled from the bulk and tends to localize into the open space of polymers such as the free volume holes in the amorphous zones and the defects in the crystalline zones. According to the orientations of the relative spins of the positron and the electron, ground state Ps (the only one of interest in condensed matter) exists as para-Ps (p-Ps, antiparallel spins) and ortho-Ps (o-Ps, parallel spins). In a vacuum, their lifetimes are 125 ps and 142 ns, respectively, and annihilation occurs into two and three photons, respectively [12]. Inside a cavity, the positron of o-Ps may interact with one of the surrounding electrons in relative singlet state and an alternative channel for annihilation is turned on. This process (‘pickoff’) reduces the o-Ps lifetime with respect to vacuum; the smaller the cavity, the more probable the process and the lower the corresponding o-Ps lifetime [13]. Such a relationship between o-Ps lifetime and size of the hole is the heart of the PALS method applied to polymers and, more in general, to soft matter. Concerning p-Ps, its lifetime is only slightly changed on passing from vacuo to matter since the intrinsic decay rate is usually much higher than the pickoff decay rate. Consequently, only the o-Ps lifetime is of interest in free volume investigations.

It is possible to quantitatively evaluate the volume of a hole by transforming o-Ps lifetime into a size, provided that the cavity, which in fact is irregularly shaped, is framed in a regular geometry by applying a suitable quantum-mechanical model. The most widely used one is due to Tao and Eldrup [14,15], which is based on the assumption of a spherical hole and produces the following relationship between the o-Ps lifetime *τ*_3_ and radius *R*:

(1) $$\tau_{3}^{-1}=\lambda_{0}\left[\frac{\Delta R}{R+\Delta R}+\frac{1}{2\pi }sen\left(2\pi \frac{R}{R+\Delta R}\right)\right]$$

where *λ_0_* ≅ 2 ns^−1^ is the annihilation rate of *o*-Ps in the presence of a high electron density and the empirical parameter ΔR (=0.166 nm [16]) accounts for the penetration of the Ps wave function into the bulk. The hole volume *v*_h_ = 4*πR*^3^/3.

The spherical geometry is certainly the simplest assumption, but it is not necessarily the most appropriate one. Unfortunately, alternative geometries have been rarely adopted to explain PALS results: ellipsoidal holes [17] in polyether ether ketone (PEEK) or cylindrical cavities in syndiotactic polystyrene [18], to quote two of the few examples. However, molecular dynamics simulations seem to indicate that non-spherical cavities can be found in some stiff chain polymers [19].

The number density of holes, *N*, is obtained from the relation:

*V_sp_* = *Nv_h_* + *V_occ_* (2)

which simply expresses the specific volume *V_sp_* as the sum of the occupied specific volume *V_occ_* and the (specific) free volume. The free volume fraction *f* can be evaluated as:

(3) $$f=\frac{Nv_{h}}{V_{sp}}$$

(we point out that this depends on the choice of the adopted hole geometry) and can be compared with *h*, which is independent of the shape of the cavities. Such a comparison may give insights on the geometry of the hole that best approximates the real shape.

Furthermore, hole models implicitly suppose that the cavity uniformly expands with temperature. Such a surmise could also prove unrealistic due to the constraints the macromolecular chains can impose on the free growth of the holes.

According to this line of reasoning, in the present work, we investigated a series of vulcanized elastomers, a butadienic rubber, an isoprenic rubber, and three of their blends. The choice was due to the fact that Srithawatpong et al. [20] investigated the free volume in 1,4 *cis*-polyisoprene, 1,2-vinyl polybutadiene, and a 50:50 miscible blend using PALS and the spherical approximation for the free volume holes. An agreement with the theoretical free volume fraction was found, but at the cost of introducing a ‘phenomenological’ occupied volume higher than the one provided by the theory. Therefore, we reconsidered the problem by abandoning the assumption of spherical cavities. We modeled the holes as cylinders that expand in an anisotropical way and showed that this innovative assumption allowed us to restore a good agreement between *h* and *f* without limiting the occupied volume to values in contrast with the theory.

## 2. Results

**Figure 1:**
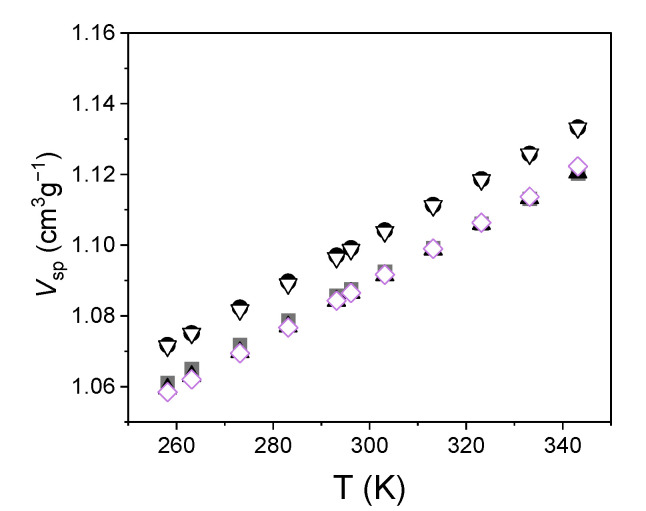
Specific volume *V_sp_* as a function of the temperature *T* in the investigated elastomers (S1: squares, S2: circles, S3: up triangles, S4: down triangles, S5: diamonds). Uncertainties are within the size of the data.

The results of the dilatometric measurements are shown in Figure 1.

The trend was perfectly linear in all cases; indeed, the correlation coefficient was always >0.995. From the data, we obtained the scaled thermodynamic parameters T* and V*, which are found in the Simha–Somcynsky equation [5]. For this purpose, we used a polynomial fit [21], which turned out to be simpler than fitting the equation; nevertheless, it produced the same numerical results. The values of the parameters are presented in Table 1.

**Figure 2:**
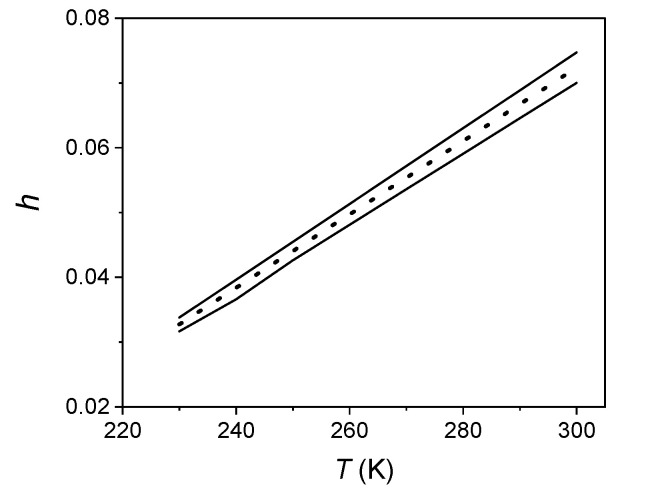
Behaviour of the free volume fraction in S3 (dotted line) as predicted by the lattice hole model. Continuous lines: uncertainties on *h*.

Their knowledge allowed us to evaluate the free volume fraction *h* predicted by the theory in the range of temperatures investigated with dilatometry. Figure 2 reports an example of the behaviour of *h* and the corresponding uncertainty.

**Figure 3:**
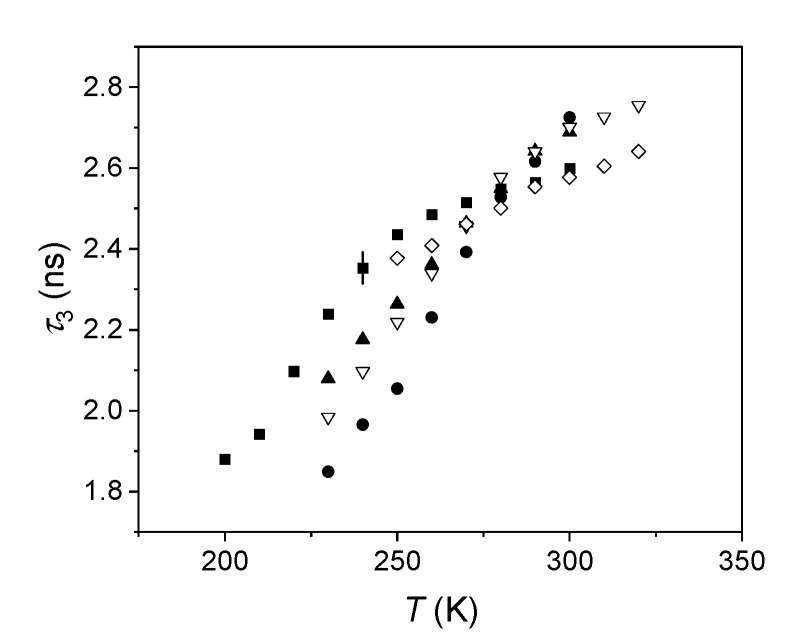
o-Ps lifetime versus the temperature in the investigated elastomers (S1: squares, S2: circles, S3: filled triangles, S4: empty triangles, S5: empty diamonds). Typical uncertainty is shown only for one lifetime for the sake of clarity.

Dilatometric results are also essential in order to evaluate the number density of holes; for this purpose, they were coupled with the PALS results, which are shown in Figure 3.

We noted a linear trend of o-Ps lifetime at increasing temperature, on a suitable range, consistently with the expansion of the free volume holes. There was also a decrease in the slope at the highest temperatures, depending on the sample, which was particularly evident for S1. This is a common feature in the investigations of polymers with PALS. This can be justified by considering that holes are not static, but dynamic: their lifetime decreases with temperature due to faster and faster molecular motions above the glass transition. It is therefore reasonable that o-Ps lifetime can be correlated to the size of the host cavity only if this last is characterized by a lifetime definitely longer than that of the atom. When the two lifetimes become comparable [22], Ps is no longer a reliable probe to obtain insights into the hole size.

**Figure 4:**
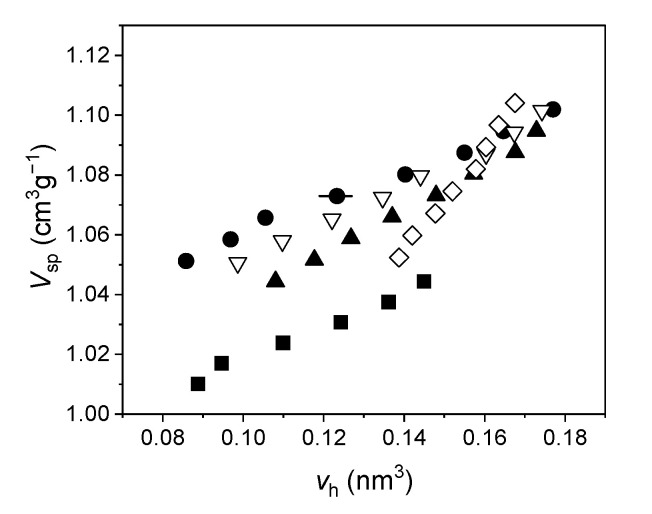
Specific volume versus the average hole volume in the investigated elastomers (S1: squares, S2: circles, S3: filled triangles, S4: empty triangles, S5: empty diamonds). Uncertainties for *V_sp_* are within the size of the symbol; typical uncertainty for *v_h_* is shown as a bar.

In the range of temperatures corresponding to the linear trend of o-Ps lifetime, we obtained the average volume of the holes, *v_h_*, versus temperature using the Tao–Eldrup Equation (1). Therefore, holes were approximated by spheres. Then, we used Equation (2) to express the specific volume as a function of the holes’ volume. The results are shown in Figure 4. Since the specific volume scales linearly with temperature, we extrapolated the specific volume at the temperatures investigated by PALS. This could be justified since in that range, no transitions occurred for the investigated elastomers.

Correlation coefficient was >0.993 for all curves, which implies that the number density of holes, *N* in Equation (2) can be considered constant in the investigated range of temperatures. Its numerical values are shown in Table 2.

**Figure 5:**
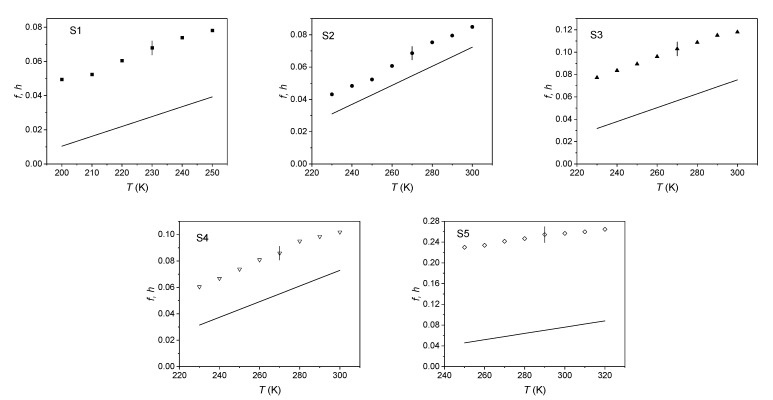
Comparison between the free volume fraction *f* as determined by positron annihilation lifetime spectroscopy (PALS) and dilatometry and *h* (continuous line).

Knowledge of *N* was used to evaluate the free volume fraction *f*, as supplied by PALS and dilatometry (Equation (3)) and to compare it with the analogous quantity *h* provided by the theory. The results are shown in Figure 5.

## 3. Discussion

**Figure 6:**
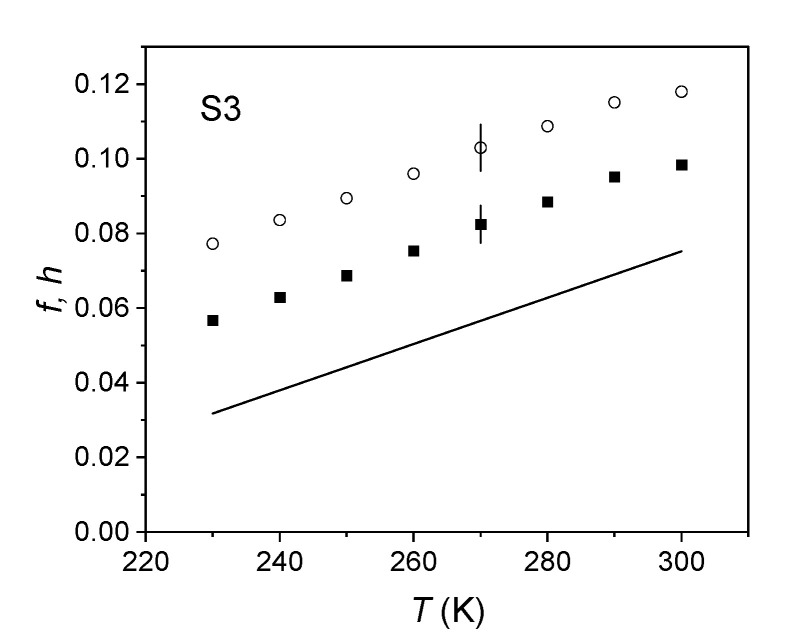
Free volume fraction in the elastomer S3 using the spherical approximation (circles) and in cylindrical geometry (squares). Continuous line: theoretical free volume fraction *h*.

The values of *f* were systematically higher than *h*. To solve the issue, we considered alternative geometries to spherical holes: cylinders [18] and cuboids [23]. Nevertheless, the results did not give an acceptable fit; an example is shown in Figure 6 for S3, using cylindrical holes with aspect ratio *q* = 0.1.

In contrast, a satisfactory fit was obtained by dropping the guess of isotropic growth of the holes with temperature. This means that the rate of growth of the cavity in one direction may be different with respect to other directions. We deemed this a physically reasonable assumption: the hole could find it more difficult to expand in given directions due to physical knots along the chain, either entanglements or crosslinks. To quantitatively assess this assumption, we modeled the holes as cylinders whose aspect ratio was not constant, but the height *s* scaled with the radius *r* according to a power law:

(4) $$\frac{s}{s_{0}}={\left(\frac{r}{r_{0}}\right)}^{p}$$

where *s*_0_ and *r*_0_ are the height and radius corresponding to the lowest temperature, respectively. Parameter *p* quantifies the anisotropy in the growth: *p* = 1 corresponds to an isotropic cylinder. The volume of the cylindrical hole results in:

(5) $$v_{h}=\pi sr^{2}=\pi q_{0}r_{0}^{1-p}r^{2+p}$$

which reduces to the usual ‘isotropic’ expression for *p* = 1. In Equation (5), *q*_0_ = *s*_0_/*r*_0_.

**Figure 7:**
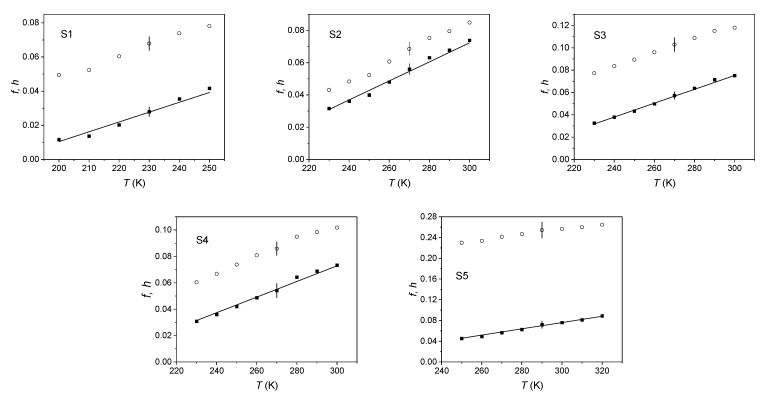
Free volume fraction in the investigated elastomers, evaluated using the spherical approximation (circles) and the anisotropic cylindrical model (squares). The continuous line represents the theoretical free volume fraction *h*.

To evaluate the free volume fraction *f* within this anisotropic geometry, we proceeded as follows. Starting from a given *q*_0_, the volume of the holes at the various temperatures was calculated according to Equation (5) by considering *p* as a free parameter. For each *q*_0_ and *p*, we used Equation (2) to obtain *N*; Equation (3) supplied *f*. Then, we evaluated the squared difference between *f* and *h* and we repeated the procedure with a new value of *p* until a minimum was reached. Finally, we changed the value of *q*_0_ and we searched for the corresponding value of *p* supplying the minimum. In this way, we found the best fit for both the parameters *q*_0_ and *p*. The results are shown in Figure 7; Table 3 displays the best fit values of *q*_0_ and *p* for the investigated elastomers. We observed an increase of *p* by increasing the amount of S1 in the blends.

According to the obtained results, we can model holes in the investigated cured elastomers in terms of flattened cylinders, which expand with temperature preferentially along the radial direction. Of course, this model is rather crude since real holes generally have an irregular shape; furthermore, we did not consider a distribution of aspect ratios due to the limited statistics of our data. Nevertheless, we deem our model reasonable by considering that in a real situation, the presence of constraints among the macromolecular chains may hinder the growth of the holes in particular directions. In fact, an isotropic growth should be an exception, rather than the rule. It should be worth comparing our results with molecular dynamics simulations on the same structures, although such work would be highly time consuming when considering the range of temperatures to be explored.

## 4. Materials and Methods

The investigated elastomers (here named Sx for simplicity) were: solution high cis polybutadiene (S1), high cis polyisoprene (S2), and their respective blends: 50% S1 + 50% S2 w/w (S3), 25% S1 + 75% S2 w/w (S4), and 75% S1 + 25% S2 w/w (S5). Every blend was mixed with 1.2 phr of dicumyl peroxide and then mold cured at high temperature.

A well-designed laboratory internal mixer was used to produce 800 g of each compound. Mixing was accomplished inside a closed chamber with “rotating kneading rotors” [24] in a type known as a Banbury mixer with a one liter mixing chamber. This is composed of tangential two wing rotors, driven at 50 RPM, with a fill factor of about 0.85; ram pressure was set at 0.4 MPa. Full cooling water was applied to the rotors and shell.

The dispersive mixing was accomplished in high-shear tapering nip regions between the rotor tips and the mixer wall. Distributive mixing occurred by transfer of material from one rotor to the other and around the mixing chamber.

For compounds S1 and S2, the raw elastomer, in chunked form, was added and the ram put down and the batch mixed for about two minutes until the temperature reached about 348 K. The ram was then raised to allow for the introduction of the curative ingredient (DCP, dicumyl peroxide). The ram was put down, and mixing was continued for another 2 min as the temperature increased to 373 K.

The batch was dumped on a two roll-mill mixer for cooling and sheeting in a slab 0.7 mm thick. Total mixing time was 4–5 min, and the final stock temperature was not higher than about 383 K. The same mixing described procedure was used to produce compounds S3, S4, and S5 by adding the related weight of the already produced S1 and S2 to the Banbury and mixing at 30 RPM until it reached the discharging temperature.

All compounds were left at room temperature for 24 h before molding in a 0.8 MPa press. Compression molds were in the form of two plates 40 × 40 cm that were removed from the press for loading and unloading. Temperature was set on the mold at 443 K and controlled by the press heating system; the mold pressure was around 0.5 MPa. The curing time was 20 min for all compounds.

Some properties of the investigated elastomers are shown in Table 4.

### 4.1. Thermal Analysis

Thermal analysis was carried out by means of a differential scanning calorimeter (DSC) 8500 Perkin Elmer, Inc. (Waltham, MA, USA), calibrated with a high purity indium standard. Samples (about 6 mg each) were encapsulated in aluminum pans and subjected, under nitrogen flux (30 cm^3^/min), to the following procedure:

(1) heating from 150 K to 323 K at 20 K/min;
(2) isothermal treatment for 1.0 min at 323 K;
(3) cooling from 323 K to 150 K at 20 K/min;
(4) isothermal treatment for 10.0 min at 150 K; and
(5) heating from 150 K to 323 K at 10 K/min.

Glass transition was evaluated from the last heating scan and the glass transition temperature *T*_g_ was obtained as the inflection point of the heat flow curve versus temperature. No other transitions were found above the *T*_g_ in the temperature range explored.

### 4.2. Positron Annihilation Lifetime Spectroscopy

The positron source, ^22^Na (activity about 4 × 10^4^ Bq), was enveloped between two Kapton^®^ foils (thickness 7.6 μm each) that were glued together. The source-support assembly was placed in a typical ‘sandwich’ configuration inside two identical cylindrical samples (diameter: 2 cm) obtained from the same batch used for dilatometric measurements. Their thickness (2 mm) was sufficient to annihilate all the positrons entering into each of them. The whole was placed inside a copper cup containing the sample and in contact with the heat exchanger of a liquid nitrogen cryostat (DN 1714 Oxford Instruments). The temperature controller granted a stability within 0.1 K. A fast-fast timing spectrometer with a resolution of about 340 ps collected the positron spectra (three spectra for each temperature). This was composed of two plastic scintillators (Pilot U, 1.5″ diameter, 1″ height) connected to Philips XP2020 photomultipliers. Conventional ORTEC instrumentation completed the spectrometer. Each spectrum contained about 10^6^ counts and was analyzed by means of the *LT* program [25] with proper correction for the positrons annihilated in the Kapton support.

### 4.3. Dilatometry

Specific volume of the investigated samples was initially determined at 296 K as the inverse of the density *ρ*, measured by means of a balance equipped with a kit for the measurement of the density based on the buoyancy method.

Specific volume measurements were carried out by means of a capillary dilatometer (bulb volume: 2.306 cm^3^, capillary length: 30 cm, capillary section: 0.0143 cm^2^) containing small pieces of the sample surrounded by mercury (purity > 99.999%, Fluka), which was used as a reference liquid. The temperature cooling runs were carried out by completely immersing the filled dilatometer in a thermostatic bath; circulating liquids were water (in the temperature range 343–283 K) and ethanol (temperature range 283–258 K). The mercury meniscus height into the capillary was determined by means of a cathetometer with digital reading. Minimal stability of the temperature was within 0.5 K. In order to obtain an average value of the specific volume at each temperature, three runs were carried out for each sample. The desired temperature was reached by cooling the sample from the previous to the new one at a rate of 0.1 K min^−1^ up to the new temperature. Then, the sample was maintained at the final temperature for 1000 s before starting the measurement, in order to ensure complete equilibrium.

## 5. Conclusions

PALS and specific volume measurements were carried out on a set of elastomers in a range of temperatures above the glass transition. By coupling the results from these two experiments, we were able to evaluate the free volume fraction and compare it to the theoretical quantity given by the lattice hole theory. Systematic differences for all samples were found by adopting a spherical geometry for the free volume holes. A similar finding was also found for non-spherical shapes for the cavities that are commonly supposed to expand isotropically with temperature. In contrast, using different expansion rates of the hole in orthogonal directions makes it possible to recover a satisfactory agreement with the theory. The introduced model of ‘anisotropic’ growth is rather simplified since it considers, for the sake of simplicity, only one kind of geometry, without taking into account a distribution of cavity sizes, mainly due to the lack of adequate statistics. Nevertheless, the main result of our work is the finding that the free volume expansion rate versus temperature is slower in some directions with respect to others, which is compatible with macromolecular segments dynamics, subjected to constraints.

The proposed approach could be proficiently exploited to determine specific volume and free volume fractions of a wide range of amorphous polymers at equilibrium, with a view to investigating several important physico-chemical properties.

## Figures and Tables

**Table 1:** Scaled thermodynamic parameters V* and T* for the investigated elastomers. Uncertainties are shown in parenthesis.

Sample	T* (K)	V* (cm^3^ g^−1^)
S1	9840 (40)	1.053 (9)
S2	9606 (39)	1.071 (9)
S3	9606 (39)	1.064 (9)
S4	9576 (39)	1.070 (9)
S5	9405 (38)	1.054 (9)

**Table 3:** Best fit values of parameters *q*_0_ and *p* for the investigated elastomers. Uncertainties are shown in parenthesis.

	S1	S2	S3	S4	S5
***q*_0_**	0.20 (0.10)	0.30 (0.10)	0.20 (0.10)	0.25 (0.10)	0.20 (0.10)
***p***	0.28 (0.10)	0.78 (0.14)	0.47 (0.13)	0.60 (0.15)	0.15 (0.09)

**Table 4:** Some thermal and mechanical properties of the investigated elastomers.

	S1	S2	S3	S4	S5
**Density** at 296 K (g/cm^3^)	0.93	0.91	0.92	0.91	0.92
**Hardness** (ShA)	64	31	48	40	58
***T_g_*** (K) DSC	166	215	215	215	214
**Stress at break** (N/mm^2^)	2.0	1.50	1.62	1.35	1.40
***G_e_*** (MPa) plateau modulus	4.5	0.85	2.6	1.4	2.3

**Table 2:** Number density of holes, *N*, in the investigated elastomers using the spherical approximation. Typical uncertainty is 6%.

	S1	S2	S3	S4	S5
*N* 10^21^ (g^−1^)	0.574	0.528	0.747	0.643	1.744
